# Dysregulation of Synaptic Signaling Genes Is Involved in Biology of Uterine Leiomyoma

**DOI:** 10.3390/genes12081179

**Published:** 2021-07-29

**Authors:** Jovan Krsteski, Mario Gorenjak, Igor But, Maja Pakiž, Uroš Potočnik

**Affiliations:** 1 Centre for Human Molecular Genetics and Pharmacogenomics, Faculty of Medicine, University of Maribor, Taborska ulica 8, 2000 Maribor, Slovenia; jkrstevski@yahoo.com (J.K.); mario.gorenjak@um.si (M.G.); 2 Department of General Gynecology and Gynecological Urology, University Clinical Centre Maribor, Ljubljanska ulica 5, 2000 Maribor, Slovenia; but.igor@gmail.com (I.B.); majapakiz@gmail.com (M.P.); 3 Laboratory of Biochemistry, Molecular Biology and Genomics, Faculty of Chemistry and Chemical Engineering, University of Maribor, Smetanova 17, 2000 Maribor, Slovenia

**Keywords:** uterine leiomyomas, *NPTX1*, *NPTX2*, *CHRM2*, *DRD2*, *CACNA1A*

## Abstract

Uterine leiomyomas are tumors, which are hormone driven and originate from the smooth muscle layer of the uterine wall. In addition to known genes in leiomyoma pathogenesis, recent approaches also highlight epigenetic malfunctions as an important mechanism of gene dysregulation. RNA sequencing raw data from pair-matched normal myometrium and fibroid tumors from two independent studies were used as discovery and validation sets and reanalyzed. RNA extracted from normal myometrium and fibroid tumors from 58 Slovenian patients was used as independent confirmation of most significant differentially expressed genes. Subsequently, GWA data from leiomyoma patients were used in order to identify genetic variants at epigenetic marks. Gene Ontology analysis of the overlap of two independent RNA-seq analyses showed that *NPTX1*, *NPTX2*, *CHRM2*, *DRD2* and *CACNA1A* were listed as significant for several enriched GO terms. All five genes were subsequently confirmed in the independent Slovenian cohort. Additional integration and functional analysis showed that genetic variants in these five gene regions are listed at a chromatin structure and state, predicting promoters, enhancers, DNase hypersensitivity and altered transcription factor binding sites. We identified a unique subgroup of dysregulated synaptic signaling genes involved in the biology and pathogenesis of leiomyomas, adding to the complexity of tumor biology.

## 1. Introduction

Uterine leiomyomas (UL), also known as uterine fibroids, are fibroid tumors that are hormone driven and originate from the smooth muscle layer of the uterine wall [1,2]. It is believed that the growth of UL is promoted by gonadal hormones, especially estrogen [3]. The tumors are characterized by deposition of extracellular matrix, which is disorganized and results in various sizes of benign neoplasms [4,5]. The estimated prevalence of UL ranges from 20 to 77% [6]. While UL are usually asymptomatic, up to 25% of women with UL may experience abdominal pain, heavy menstrual bleeding, increased risk of miscarriage or infertility [7]. The only curative treatment is hysterectomy, as non-surgical treatment options do not exist [8]. New genetic techniques have provided new insights into the pathogenesis of UL, with stratification into four main subtypes: mutations of mediator of transcription subunit 12 (*MED12*), fumarate hydratase (*FH*), high mobility group AT-hook 2 (*HMGA2*) translocations and collagen gene deletions [9]. It has been found that approximately 70% of patients with UL carry mutations in exon 2 of *MED12* [10]. Additionally, recent work has also revealed subtype-specific gene expression profiles, which supports the idea of different mechanisms of leiomyoma pathogenesis [11]. Furthermore, growth factors and their cognate receptors also play an important role in the pathogenesis of UL [12]. It was clearly shown that alterations in over-expression of *TGF-β* may contribute to the growth of UL [13,14]. TGF-β stimulation increases extracellular matrix protein production and simultaneously decreases degradation of the matrix in UL [15]. Recent state-of-the-art integrative approaches have identified a wide genetic and epigenetic etiology underlying UL. Integration of transcriptomic and epigenetic changes has allowed for the identification of differential transcription factor occupancy, differential enhancer engagement consisting of histone acetylation, and altered enhancer–promoter contact rewiring as key events in UL gene dysregulation and differential expression [16]. Moreover, the regulatory potential of altered DNA methylation driving UL development has also been shown [17]. Additionally, common loci between UL and other gynecological diseases or phenotypes have also been identified by whole-genome association studies (GWAs) [18,19], implicating a complex background in the etiology of UL and insinuating the interplay of genetic variants and epigenetic expression regulation.

As recent approaches highlight epigenetic malfunctions as important mechanisms of gene dysregulation, we aimed to use our previously published transcriptomic–genomic integration approach [20,21] to identify additional genetic–epigenetic interplay regions involved in the formation and biology of UL. For that, we used the combination and integration of publicly available data from three previously published independent studies and our own cohort. Publicly available RNA sequencing (RNA-seq) raw data reads from pair-matched normal myometrium and fibroid tumors from two independent studies [16,17] were used as discovery and validation sets in the present study. Subsequently, RNA extracted from normal myometrium and fibroid tumors from our own Slovenian cohort was used as independent confirmation of identified genes, and meta-analysis GWA summary [18] was used in order to identify genetic variants at epigenetic marks.

## 2. Materials and methods

### 2.1. Subjects

We enrolled 36 Slovenian patients with clinically diagnosed UL who had undergone surgical treatment at the Department of General Gynecology and Gynecological Urology (University Medical Centre Maribor, Maribor, Slovenia). Out of 36, 14 women presented with solitary and 22 with multiple uterine fibroids. RNA was extracted from solitary tumors and from one tumor of patients with multiple UL. Clinical data are presented in Table 1. Additionally, 22 patients who underwent surgical treatment for non-UL-related conditions (with pelvic organ prolapse) were enrolled as controls in order to extract RNA from normal myometrium. The mean age of enrolled women was 43.3 ± 6.5 years for cases and 60.6 ± 11.5 years for controls. The study protocol was approved by the National Medical Ethics Committee and the Institutional Review Board (KME 43/10/15). Written informed consent was obtained from all enrolled subjects.

### 2.2. RNA Sequencing Analysis

RNA-seq analysis was performed using publicly available pair-matched paired-end raw datasets from SRP166862 and SRP217468 [17] and SRP188330 [16] from previously published studies. Datasets SRP166862 and SRP217468 were used as discovery and validation sets in the study performed by George and colleagues [17] and were merged to obtain the discovery dataset in the present study. The discovery dataset consisted of 15 matched normal myometrium and fibroid tumors. If multiple tumors were present, samples from other UL were discarded from the analyses. Dataset SRP188330 was used as the validation set in the present study and also consisted of 15 matched normal myometrium and fibroid tumors. Both datasets were analyzed independently using the R 4.0.2 environment (R Core Team 2020, Vienna, Austria). Paired-end reads were mapped to the hg19 reference genome and assigned to genomic features using Rsubread 2.2.4 R package and featureCounts [22,23]. Counts per million (CPMs) were estimated using edgeR 3.30.3 R package [24] and low expressed genes were filtered out based on CPMs corresponding to read counts of 10. Retained genes were normalized using the trimmed mean of M values method [25]. Mean–variance modeling at the observational level transformation (VOOM) was applied [26], and differential expression of fibroid tumors relative to normal myometrium was estimated using models and empirical bayes implemented in limma 3.44.3 R package [27] and using blocking to adjust for paired samples. Differential expression was considered for genes with *q* value < 0.05 and loget > 2 or <−2 (loget = log_2_(fibroid/normal)).

### 2.3. Gene Ontology Analysis

Gene Ontology analysis was performed using the software package CytoScape 3.8.1 [28] with integrated application ClueGO v2.5.7 [29]. ClueGO analysis was performed using the following parameters and selected options: Ontology/Pathways selected: Biological Process, Cellular Component and Molecular Function Evidence selected: only All_Experimental.

### 2.4. Extraction of RNA

RNA was extracted from 25 to 30 mg of fibroid tumors and normal myometrium using a miRNeasy mini kit (QIAGEN, Germantown, ML, USA) after manual homogenization. Purity and concentration of nucleic acids were determined using Synergy 2 spectrophotometer (BioTek, Winooski, VT, USA), and integrity of RNA was checked using agarose gel electrophoresis.

### 2.5. Validation Using RT-qPCR

A total of 1 µg of mRNA was transcribed into cDNA using a high-capacity cDNA reverse transcription kit (Thermo Fisher, Waltham, MA, USA). Nucleotide sequences of target genes *NPTX1*, *NPTX2*, *DRD2*, *CHRM2* and *CACNA1A* were obtained from the NCBI Nucleotide database (www.ncbi.nlm.nih.gov/nuccore/, accessed on 15 March 2021), and isoform non-specific primers were hand-picked using IDT OligoAnalyzer Tool (eu.idtdna.com/calc/analyzer, accessed on 15 March 2021). Reference genes primers for *ACTB* and *B2M* were obtained from a previous study [30]. Primer sequences and accession numbers are summarized in Table 2. Primers were synthesized by Sigma (Merck, Darmstadt, Germany). Reverse transcription quantitative polymerase chain reaction (RT-qPCR) gene expression experiments were carried out using LightCycler 480 SYBR Green I Master Mix and a LightCycler 480 real-time thermocycler (Roche, Basel, Switzerland). An amount of 2 µL of 10-fold diluted cDNA (5 ng/µL) was used as a template. Efficiency was >90% for all primer pairs, and specificity of amplification was estimated using melting curves for each sample after each run. Raw C_T_ values were obtained from three run-independent technical replicates for each sample. Geometric averaging of reference genes was used for normalization, and relative expression was calculated using the 2^−ΔΔCt^ method [31]. Statistical analysis was performed using linear 2^−ΔCt^ calculation and binomial generalized linear models adjusted to age in the R environment.

### 2.6. Correlation Analyses and Machine Learning Prediction Value Estimation

In order to perform correlation analyses, raw counts from publicly available RNA-seq data were transformed to transcripts per million (TPM). Both discovery and validation datasets were first merged and divided into normal myometrium and fibroid tumors datasets in order to obtain 30 samples per set. Correlation analyses were performed using the 4.0.2 environment (R Core Team 2020, Vienna, Austria) and using PerformanceAnalytics 2.0.4 R package (github.com/braverock/PerformanceAnalytics). Additionally, all five genes were further assessed using randomForest 4.6-14 [32] R package and receiver operating characteristics analysis using *Proc* [33] R package. The assessment was made independently for obtained RNA-seq TPM data and obtained RT-qPCR data.

### 2.7. Integration to Meta-GWAs and In Silico Functional Analysis

Uterine leiomyoma GWA data were obtained from publicly available summary statistics of uterine leiomyoma meta-analysis of cohorts of the Women’s Genome Health Study, UK Biobank, Queensland Institute of Medical Research, and the North Finnish Birth Cohort of white European ancestry (www.ebi.ac.uk/gwas/studies/GCST009158, accessed on 5 May 2021) [18]. Summary statistics included summaries for 11,464,556 variants. All variants ranging ±100 kb from previously identified differentially expressed genes were extracted and further analyzed. Functional analyses were conducted using HaploReg v4.1 [34] and GTEx Portal [35]. Regional Manhattan plots were constructed using LocusZoom [36].

## 3. Results

### 3.1. RNA Sequencing and Differential Expression

**Figure 1:**
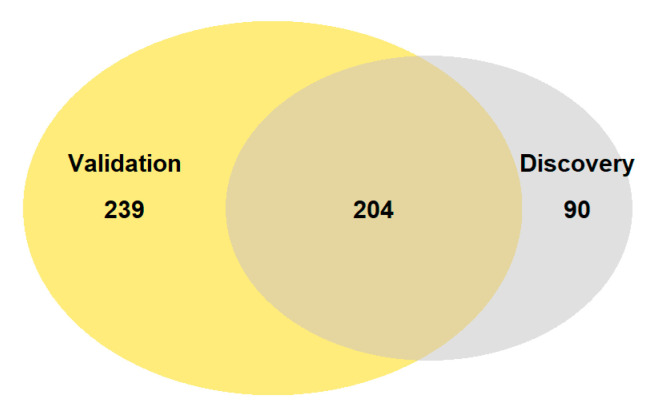
Overlap of significantly expressed genes of discovery and validation RNA-seq analysis.

Using available pair-matched raw datasets (SRP166862 and SRP217468; merged and used as discovery [17] and SRP188330; used as validation) [16] from previously published studies, we performed our own RNA-seq analysis using the aforementioned pipeline in the R environment. The discovery dataset was first filtered according to *q* value < 0.05 and loget > 2 or <−2, where 294 significantly differentially expressed genes were observed in fibroid tumors relative to normal myometrium (Table S1). Subsequently, the same RNA-seq pipeline was applied to the validation cohort, where 443 significantly differentially expressed genes were observed in fibroid tumors relative to normal myometrium, using the same filtering thresholds (Table S1). The results of the validation dataset RNA-seq analysis confirmed 204 significantly differentially expressed genes from the discovery dataset (Figure 1).

### 3.2. GO Analysis

**Figure 2:**
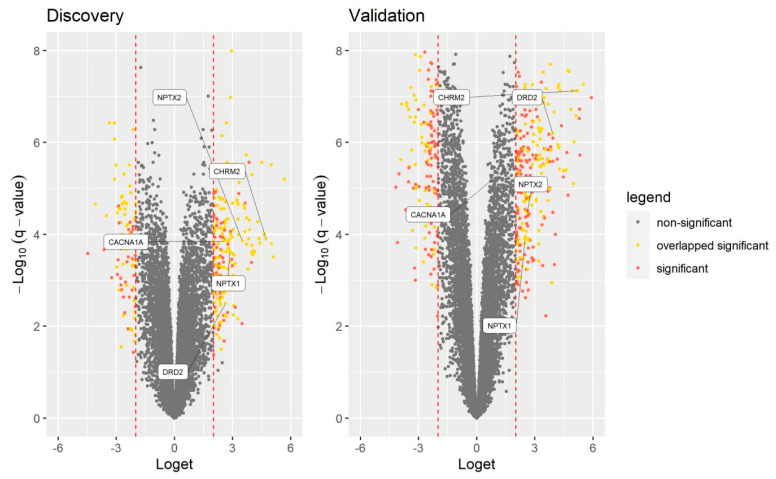
Volcano plots with overlapped and selected genes.

Subsequent Gene Ontology analysis of 204 genes confirmed on the validation dataset showed that 15 significantly enriched terms were listed after Bonferroni correction (Table 3). It was observed that *NPTX1* and *NPTX2* genes were listed at eight enriched terms, followed by *DRD2* and *CHRM2*, which were listed at seven enriched terms. Interestingly, the *CACNA1A* gene was listed at four enriched terms where all four aforementioned genes were also present. All five genes were significantly upregulated in both the discovery and validation datasets (Figure 2). Based on that observation, *NPTX1*, *NPTX2*, *DRD2*, *CHRM2* and *CACNA1A* genes were considered for an additional validation using RT-qPCR.

### 3.3. RT-qPCR Validation of GO Identified Genes

**Figure 3:**
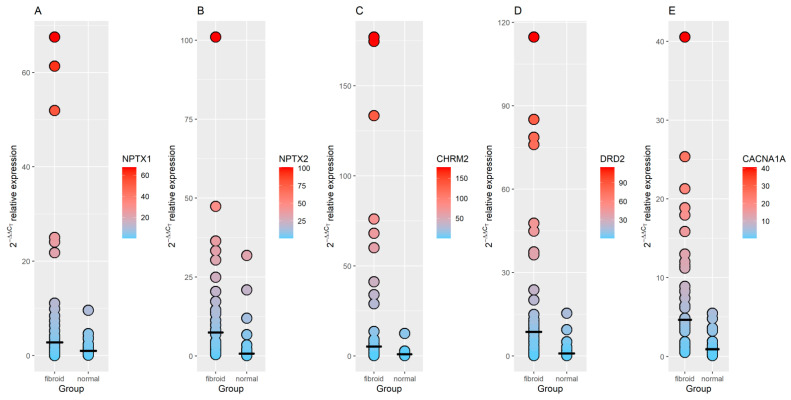
RT-qPCR results of selected genes. (**A**): *NPTX1*; (**B**): *NPTX2*; (**C**): *CHRM2*; (**D**): *DRD2*; (**E**): *CACNA1A*; fibroid: fibroid tumor; normal: normal myometrium.

*NPTX1*, *NPTX2*, *DRD2*, *CHRM2* and *CACNA1A* were selected for an additional validation using RT-qPCR. All five genes were upregulated in both discovery and validation datasets (Table 4). Subsequent RT-qPCR analysis was performed using RNA extracted from Slovenian patients, from 22 unmatched normal myometrium and 36 unmatched fibroid tumors. All five genes were proven to be statistically significantly upregulated in fibroid tumors relative to normal myometrium (Table 4; Figure 3). Moreover, the gene expression of *NPTX1*, *NPTX2*, *CHRM2*, *DRD2* and *CACNA1A* was significantly upregulated throughout both RNA-seq and RT-qPCR analyses. Raw RT-qPCR C_T_ values are provided in Table S2.

### 3.4. Between-Gene Regulation of the Identified Genes and Prediction Value Estimation

**Figure 4:**
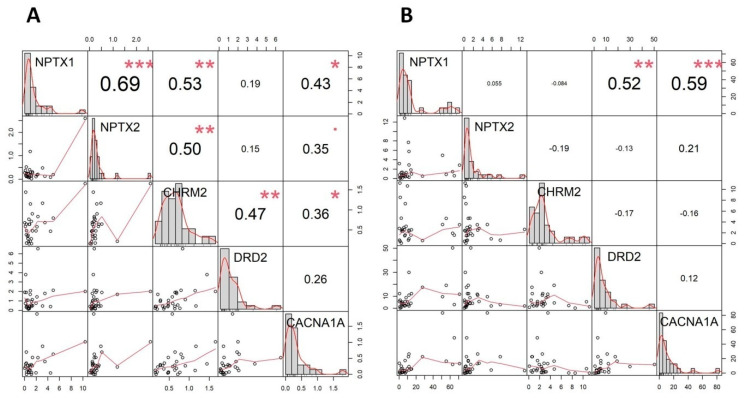
Correlation matrices. ***: significant at 0.001; **: significant at 0.01; *: significant at 0.05. (**A**) normal myometrium; (**B**) fibroid tumor.

In order to assess the relationships between the identified genes, we performed correlation analyses. For that, discovery and validation RNA-seq datasets containing raw counts were merged, TPMs were calculated, and datasets were split into normal myometrium and fibroid tumor datasets. Using normal myometrium data, the correlation results showed an extensive interplay between all five genes (Figure 4A), whereas using fibroid tumor data, the correlation was retained only for *NPTX1* and *CACNA1A* (Figure 4B). Moreover, in normal data, the correlation between *NPTX1* and *DRD2* was not observed, but in fibroid tumor data, the correlation between *NPTX1* and *DRD2* was observed. Subsequently, calculated TPMs and RT-qPCR results were used independently to assess the prediction value of selected genes using machine learning approach. For both RNA-seq and RT-qPCR obtained data, the Random Forest machine learning algorithm further confirmed the involvement of the five genes in uterine leiomyoma. For both datasets, the accuracy of prediction was 100% and AUC: 1.

### 3.5. Integration into Uterine Leiomyoma Meta-GWAs and Functional Analysis

**Figure 5:**
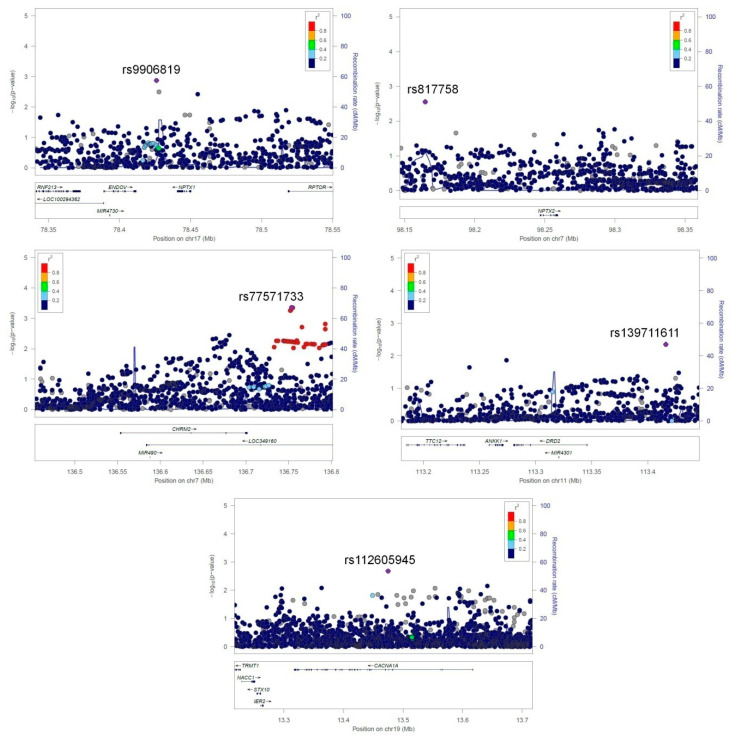
Regional Manhattan plots for selected gene regions from meta-analysis summary.

Subsequently, validated and confirmed expression results were integrated using previously published meta-analysis results achieved by Gallagher and colleagues [18]. We analyzed single nucleotide variants from meta-analysis summary statistics ranging ±100 kb from previously identified differentially expressed genes, and we extracted the most significant signals at each region (Table 5). For genes *NPTX1*, *NPTX2*, *CHRM2*, *DRD2* and *CACNA1A*, we found significant 3′ downstream variant rs9906819, 5′ upstream variant rs817758, 3′ downstream variant rs77571733, 5′ upstream variant rs139711611 and intronic rs112605945 variant, respectively (Figure 5). No eQTL data were available for SNPs and selected genes. Using HaploReg, rs9906819 is listed as a genic enhancer by the Core 15-state model and 25-state model, and at H3K4me1_Enh and H3K27ac_Enh epigenetic chromatin state marks in various cells, there are also female skeletal muscle cells. Additionally, DNase hypersensitivity in fetal and psoas muscle is listed at rs9906819, and ChIP-seq evidence of NRSF binding exists at rs9906819. The PBX3 putative transcription factor binding motif is also listed as altered by the variant. SNP rs817758 is listed only at the H3K4me3_Pro epigenetic mark in lungs and spleen, and the YY1 putative transcription factor binding motif is altered by the variant. SNP rs77571733 is listed at H3K4me1_Enh in muscle, mesenchymal stem cells and lung fibroblasts, and POU2F2 putative transcription factor binding motif is listed as altered by the variant. SNP rs139711611 is listed at the H3K4me1_Enh epigenetic mark in mesoderm cultured cells and mammary epithelial cells. DNase hypersensitivity is also listed at rs139711611 in iPSCs. rs112605945 is listed as a genic enhancer by the Core 15-state model and 25-state model. DNase hyperactivity is also listed at the variant by 25-state model. The variant is also listed at H3K4me1_En, H3K4me3_Pro, H3K27ac_Enh and H3K9ac_Pro epigenetic chromatin state marks. CTCF, HNF4, NRSF, SP1 and Sin3Ak-20 putative transcription factor binding motifs are also listed as altered by the variant.

## 4. Discussion

The biology of uterine leiomyoma is still not well understood, and recent state-of-the-art integrative studies have shown that in addition to already established genetic stratification into four main subtypes, epigenetic malfunctions are recognized as important mechanisms of gene dysregulation in UL pathogenesis. Using the integration of transcriptomic and genetic data, the present study identified synaptic signaling genes *NPTX1*, *NPTX2*, *CHRM2*, *DRD2* and *CACNA1A*, a unique subgroup of dysregulated genes in the biology of UL. To the best of our knowledge, this is the first time that synaptic signaling genes were observed to be associated with UL. GO analysis of the overlap of two independent RNA-seq analyses showed that *NPTX1*, *NPTX2*, *CHRM2*, *DRD2* and *CACNA1A* were listed as significant for several enriched GO terms. These genes were subsequently validated using RT-qPCR in our own cohort consisting of 36 and 22 Slovenian patients and controls, respectively. The validation using Slovenian samples additionally confirmed the aforementioned genes. We observed that all five genes show significant upregulation in fibroid tumors relative to normal myometrium. However, Slovenian samples were not pair-matched, which presents a limitation of the study. Moreover, the present study also showed between-gene dysregulation when comparing the gene–gene interactions between normal myometrium and fibroid tumor. Correlation analysis has clearly shown that there is a shift in gene–gene regulations in fibroid tumors in terms of lost interplay. Moreover, based on correlation figures, it is clearly evident that the expression of the aforementioned genes is upregulated in fibroid tumors, ranging above 10 TPMs in comparison with normal myometrium, which ranges up to but not above 10 TPMs. The involvement of *NPTX1*, *NPTX2*, *CHRM2*, *DRD2* and *CACNA1A* was additionally confirmed using a machine learning approach, which also confirmed the involvement of these genes in the biology of UL in two independent datasets obtained from RNA-seq analyses and an RT-qPCR experiment. As interesting as this finding may seem, there is evidence of association between the aforementioned genes and tumorogenesis in some cancers. *NPTX1* (neuronal pentraxin 1) is a member of the pentraxin family and can bind various ligands, such as bacteria and also chromatin [37]. Molecular studies also reveal that NPTX1 functions by regulating both Nodal ligands and bone morphogenetic proteins (BMP) signaling via binding to TDGF1, which regulates pluripotency and neural differentiation [37]. Both Nodal ligands and BMP are also members of the TGF-β family of ligands [38], which is in accordance with previous findings where *TGF-β* was considered a key player gene in UL [39] and further extends previously identified pathways to synaptic signaling. Furthermore, NPTX1 was also identified as a novel epigenetic regulator that was associated with prognosis in lung cancer [40]. *NPTX2* (neuronal pentraxin 2) is also a member of the highly conserved pentraxin protein group and was previously associated with neurodegenerative diseases [41]. Additionally, it was also previously observed that *NPTX2* hypermethylation inhibits cell cycle arrest and apoptosis in gastric cancers via p53 suppression [42], which in turn suggests a possible association of *NPTX2* with epigenetic regulation. Synaptic receptors *CHRM2* (cholinergic receptor muscarinic 2) and *DRD2* (dopamine receptor D2) have also been listed at significantly enriched terms in GO analysis. To the best of our knowledge, *CHRM2* is not associated with conditions or functions that could put the gene on the radar in the development of UL pathogenesis. However, expression of *CHRM2* is co-regulated with the expression of *DRD2* in healthy myometrium but not in fibroid tumors, where correlation between these two genes has not been observed. In contrast to *CHRM2*, *DRD2* has previously been associated with UL. It has been shown that *DRD2* codon 313(*)T-related genotypes/alleles are associated with the presence of UL [43]. Interestingly, it was observed that the *CACNA1A* gene was listed at four enriched terms, where all four aforementioned genes were also present. *CACNA1A* gene encodes the voltage-dependent P/Q-type calcium channel subunit α-1A and is widely expressed throughout the central nervous system. *CACNA1A* has previously been associated with a wide spectrum of neurological disorders [44]. Additionally, with epigenetic exploration, it was shown that methylation of *CACNA1A* is one of the markers for irradiation efficacy in oropharyngeal cancer [45]. Furthermore, *CACNA1A* has also been shown to have a high promoter methylation status in ovarian clear cell adenocarcinoma [46]. Methylation of *CACNA1A* is also associated with triple-negative breast cancer [47]. The aforementioned epigenetic associations of *CACNA1A* support the gene’s involvement in epigenetics of tumorogenesis. Interestingly, a previous study demonstrated that innervation of the uterus is involved in multiple pathophysiological processes and suggests that autonomic innervations together with interstitial telocytes are involved in the microenvironmental imbalance of UL [48]. Moreover, the difference in adrenergic and cholinergic innervation between normal myometrium and fibroid tumors demonstrates the pivotal role of the neuronal component in the formation of UL [48]. These findings are further supported by the unique subset of genes identified by the present study. Subsequently, in order to further address and identify additional genetic–epigenetic landmarks involved in the formation of UL, we integrated a previously published genome-wide association meta-analysis summary [18] with identified gene regions ranging ±100 kb from the genes. We selected the most significant SNPs in these regions and extracted four near gene variants (rs9906819, rs817758, rs77571733 and rs139711611) and one intron variant rs112605945 harboring in *CACNA1A*. None of the five variants was previously mentioned to be associated with any phenotype. Using HaploReg [34], we assessed the potential interplay between these variants and epigenetic landmarks. All five variants were listed at epigenetic chromatin state marks predicting enhancer and promoter histone modification. SNPs rs9906819 and rs139711611 were also listed as genic enhancers. Moreover, rs9906819, rs817758, rs77571733 and rs112605945 were flagged as altering a putative transcription factor binding motif. Additionally, rs9906819, rs139711611 and rs112605945 were listed at a DNase hypersensitivity chromatin structure. By establishing global chromatin states, histone modifications influence gene expression [49]. Histone residues can be acetylated or methylated [50], and acetylation is considered as a hallmark of active transcription [51]. Thus, the locations of the selected variants may help to elucidate the upregulation and dysregulation of the selected genes in UL tissue. In silico functional analysis using HaploReg clearly showed that the locations of the selected SNPs and corresponding effect alleles may influence the expression of the aforementioned genes. Moreover, these findings warrant a chromatin immunoprecipitation (ChIP) assay, as they are listed at specific chromatin states, protein binding sites and regulatory motifs. We hypothesize that the effect alleles of the selected variants may cause histone modifications in favor of upregulation of nearby genes and may additionally alter transcriptions binding motifs, promoting gene expression and thus the formation of the fibroid tumors. However, the role of the synaptic signaling genes in the biology of UL must be further elucidated. We believe that integration of independent studies and -omics may help to identify new signals, which are otherwise masked due to strong statistics signals obtained through homogenous sampling.

## 5. Conclusions

In summary, the present study identified a unique subgroup of dysregulated synaptic signaling genes in the biology of UL, adding to the complexity of the tumor biology. These findings may increase our understanding of the broad molecular interplay of signaling pathways and neuronal components in the formation of UL and further support epigenetic regulation as an important mechanism of uterine leiomyoma disease pathogenesis.

## Figures and Tables

**Table 1:** Clinical data of enrolled patients with UL.

Data	Values
Age mean (95% CI)	43.3 (40.6–45.4)
Positive familial history (%)	17.7
Menarche mean (95% CI)	12.6 (12.1–14.1)
Pregnancy mean (95% CI)	3 (2.5–3.5)
Parity mean (95% CI)	1.9 (1.6–2.2)
Miscarriage mean (95% CI)	0.6 (0.2–1.0)
Oral contraceptives (%)	60
Progestin therapy (%)	16
BMI mean (95% CI)	26.3 (25.0–28.5)

**Table 2:** Primer sequences and accession numbers.

GENE	ACCESSION	FW 5′ to 3′	RV 5′ to 3′
*NPTX1*	NM_002522.4	GTGGCAGTGGCGAGAACT	GGTCCCAGATGTTGAAGTGG
*NPTX2*	NM_002523.3	CAGGACGGAGAGAAGCTG	AGTGGCATCAAACCTACCC
*CHRM2*	NM_001006630.2	CTATCAACCCTGCCTGCTAT	ACCTTGTAGCGCCTATGTTC
*DRD2*	NM_000795.4	TCCCAGCAGAAGGAGAAGAA	TGTTCAGGATGTGTGTGATGAA
*CACNA1A*	NM_000068.4	TTGTGGTGTTCCCCTTCTTC	ACATGCGGTACTGGAAGCTC

**Table 3:** Significant GO terms for 204 confirmed genes.

GO ID	GO TERM	ONTOLOGY	BONFERRONI *p*	GENES
GO:0098644	complex of collagen trimers	Cellular component	0.004	*COL11A1*, *COL2A1*, *COL4A4*
GO:0009308	amine metabolic process	Biological process	0.004	*ATCAY*, *HDC*, *INMT*, *TDO2*
GO:0098960	postsynaptic neurotransmitter receptor activity	Biological process	0.006	*CHRM2*, *DRD2*, *NPTX1*, *NPTX2*
GO:0032835	glomerulus development	Biological process	0.015	*BMP7*, *CD24*, *COL4A4*
GO:0001822	kidney development	Biological process	0.020	*BMP7*, *CD24*, *COL4A4*, *OSR1*, *STRA6*
GO:0099565	chemical synaptic transmission, postsynaptic	Biological process	0.025	*CHRM2*, *DRD2*, *NPTX1*, *NPTX2*
GO:0072001	renal system development	Biological process	0.025	*BMP7*, *CD24*, *COL4A4*, *OSR1*, *STRA6*
GO:0001655	urogenital system development	Biological process	0.027	*BMP7*, *CD24*, *COL4A4*, *OSR1*, *STRA6*
GO:0098916	anterograde trans-synaptic signaling	Biological process	0.027	*CACNA1A*, *CHRM2*, *DRD2*, *NPTX1*, *NPTX2*
GO:0007268	chemical synaptic transmission	Biological process	0.027	*CACNA1A*, *CHRM2*, *DRD2*, *NPTX1*, *NPTX2*
GO:0099537	trans-synaptic signaling	Biological process	0.030	*CACNA1A*, *CHRM2*, *DRD2*, *NPTX1, NPTX2*
GO:0030594	neurotransmitter receptor activity	Molecular function	0.031	*CHRM2*, *DRD2*, *GRIA2*, *NPTX1*, *NPTX2*
GO:0044106	cellular amine metabolic process	Biological process	0.033	*ATCAY*, *HDC*, *TDO2*
GO:0099536	synaptic signaling	Biological process	0.034	*CACNA1A*, *CHRM2*, *DRD2*, *NPTX1, NPTX2*
GO:0010469	regulation of signaling receptor activity	Biological process	0.034	*CRHBP*, *NPTX1*, *NPTX2*

**Table 4:** RNA-seq and RT-qPCR results of selected genes.

	Discovery		Validation		RT-qPCR	
GENE	LOGET	*q* VALUE	LOGET	*q* VALUE	FC	*p* VALUE
*NPTX1*	2.84	2.3 × 10^−4^	2.49	1.3 × 10^−4^	2.55	0.013
*NPTX2*	3.50	1.4 × 10^−4^	2.60	3.4 × 10^−5^	7.34	0.015
*DRD2*	2.63	3.2 × 10^−3^	3.91	6.4 × 10^−7^	6.08	0.016
*CHRM2*	4.72	1.3 × 10^−4^	5.19	7.5 × 10^−8^	5.77	0.018
*CACNA1A*	2.55	1.4 × 10^−4^	2.18	2.1 × 10^−6^	4.68	0.024

Loget: Log_2_FC fibroid tumors relative to normal myometrium; FC: fold-change fibroid tumors relative to normal myometrium.

**Table 5:** Most significant SNPs in selected gene regions.

GENE	SNP	CONS	REF	ALT	EFFECT SIZE	ST ERROR	*p* VALUE	N
*NPTX1*	rs9906819	3’ downstream	A	C	0.036	0.011	0.0013	244324
*NPTX2*	rs817758	5’ upstream	T	C	0.085	0.029	0.0028	226299
*CHRM2*	rs77571733	3’ downstream	A	G	−0.172	0.049	0.0004	238961
*DRD2*	rs139711611	5’ upstream	A	C	−0.151	0.053	0.0044	238961
*CACNA1A*	rs112605945	intron variant	T	C	0.078	0.026	0.0021	244324

CONS: consequence; REF: reference allele; ALT: alternative allele; ST ERROR: standard error of meta-analysis; N: number of individuals at each observation.

## Data Availability

Publicly available datasets were analyzed in this study. This data can be found here: (https://www.ncbi.nlm.nih.gov/sra/SRP188330; https://www.ncbi.nlm.nih.gov/sra/SRP166862; https://www.ncbi.nlm.nih.gov/sra/SRP217468; www.ebi.ac.uk/gwas/studies/GCST009158 accessed on 5 May 2021). The data presented in this study are also available in Table S2.

## Supplementary Materials

The following are available online at https://www.mdpi.com/article/10.3390/genes12081179/s1, Table S1: RNA-seq complete differential expression from discovery and validation cohort; Table S2: Raw Ct values from RT-qPCR.
